# The relationship between sarcopenia and survival at 1 year in patients having elective colorectal cancer surgery

**DOI:** 10.1007/s10151-019-02072-0

**Published:** 2019-09-05

**Authors:** D. R. Dolan, K. A. Knight, S. Maguire, S. J. Moug

**Affiliations:** 1 School of Medicine, University of Glasgow, Glasgow, UK; 2 Department of Surgery, Royal Alexandra Hospital, Corsebar Road, Paisley, PA2 9PN UK

**Keywords:** Colorectal cancer, Sarcopenia, One-year survival

## Abstract

**Background:**

Colorectal cancer remains a common cause of cancer death in the UK, with surgery being the mainstay of treatment. An objective measurement of the suitability of each patient for surgery, and their risk–benefit calculation, would be of great utility. We postulate that sarcopenia (low muscle mass) could fulfil this role as a prognostic indicator. The aim of this study was to determine the relationship between sarcopenia and long-term outcomes in patients undergoing elective bowel resection for colorectal cancer.

**Methods:**

One hundred and sixty-three consecutive patients who had elective curative colorectal resection for cancer were eligible for inclusion in the study. Psoas muscle mass was assessed on preoperative computed tomography scan at the level of the L3 vertebra and standardised for patient height (total psoas index, TPI). Sarcopenia (low muscle mass) was defined as < 524 mm^2^/m^2^ in males and 385 mm^2^/m^2^ in females. In addition to clinical–pathological parameters, postoperative complications were recorded and patients were followed up for mortality for 1 year after surgery.

**Results:**

Sarcopenia was present in 19.6% of the study participants and was significantly related to body mass index (*p* = 0.007), 30-day mortality (*p* = 0.042) and 1-year mortality (*p* = 0.046). In univariate analysis, American Society of Anesthesiologists grade (*p* = 0.016), tumour stage (*p* = 0.018) and sarcopenia (*p* = 0.043) were found to be significant independent predictors of 1-year mortality.

**Conclusions:**

This study has found sarcopenia to be prevalent in patients with colorectal cancer having elective surgery. Independent of age, sarcopenia was associated with poorer 30-day mortality and survival at 1 year. Measurement of muscle mass preoperatively could be used to stratify a patient’s risk, allowing targeted strategies such as prehabilitation, to be implemented to modify sarcopenia and improve long-term outcomes for patients.

## Introduction

Colorectal cancer is the fourth most common cancer in the UK, with over 41,000 new diagnoses in 2014 [1]. With incidence increasing with age, and the ageing population expanding, there is a clinical need for new prognostic knowledge-aided decision making to improve long-term outcomes [2, 3]. One potential prognostic marker is sarcopenia. Sarcopenia was defined in 2010 as ‘a syndrome characterised by progressive and generalised loss of skeletal muscle mass and strength’ (the European Working Group on Sarcopenia in Older People (EWGSOP) [4]. There are various techniques available to measure sarcopenia, varying from simple to complex, but computed tomography (CT) measurement of the psoas muscle, with established cutoffs defining sarcopenia, is a widely accepted technique [4].

There have been numerous studies that have assessed the relationship between sarcopenia and surgical outcomes in patients having various intra-abdominal operations, including radical cystectomy, liver transplantation and emergency general surgery, with their findings pointing towards an association between sarcopenia and mortality [5–8].

Studies specifically analysing the influence of sarcopenia on postoperative elective colorectal cancer patients have found that the presence of sarcopenia results in an increased risk of postoperative complications, increased length of hospital stay and increased cost of care [9–15]. A study published in 2019 showed that sarcopenia is highly predictive of serious postoperative complications in colorectal cancer patients [15]. In contrast, there are only a few studies focusing on the relationship between sarcopenia and long-term mortality [16–19]. Although all suggested a negative influence of sarcopenia on 1-year survival, drawing conclusions is difficult due to the different patient populations studied and varying methodologies.

The primary aim of this study was to determine the relationship between preoperative sarcopenia and mortality at 1 year in patients who had elective colorectal cancer resection.

## Materials and methods

### Data sources and study population

The study was registered with the NHS Greater Glasgow and Clyde Clinical Effectiveness Department (August 2016). Analysis was undertaken of data extracted from the prospective enhanced recovery after surgery (ERAS) database at the Royal Alexandra Hospital, Paisley, for the period January 2015–December 2016. This database has been set up as a part of the National Enhanced Recovery Colorectal Initiative (NERCI) supported by the Whole System Patient Flow Improvement Programme as part of the Scottish Government’s Health Performance and Delivery Directorate. Briefly, data from all elective colorectal surgery (both benign and malignant pathology) are submitted from each surgical unit in Scotland every month with regular feedback after central analysis of the data.

Inclusion criteria extended to any patient who had elective colorectal resection with curative intent, where pathology had confirmed colonic or rectal adenocarcinoma. Stage 4 patients with resectable liver and lung metastases, as adjudged by the relevant clinical specialists, were included. Patients were excluded if: they had received neo-adjuvant therapy; had surgery to treat any condition other than colorectal cancer; had widespread unresectable metastatic disease; had surgery as an emergency; if no preoperative CT scan was available; or if their most recent CT scan was performed more than 4 months before surgery.

Demographic, anthropometric and clinical data including height, weight, body mass index (BMI) [weight (kg)/height (m)^2^], American Society of Anesthesiologists (ASA) score, operative procedure, American Joint Committee on Cancer (AJCC) TNM stage [20], length of hospital stay (days), postoperative complications and grade [21] and re-admission to the hospital within 30 days were analysed. In addition, patients’ electronic records were followed up for mortality for 1 year after the date of surgery.

### Sarcopenia measurement: CT scan analysis

**Fig. 1:**
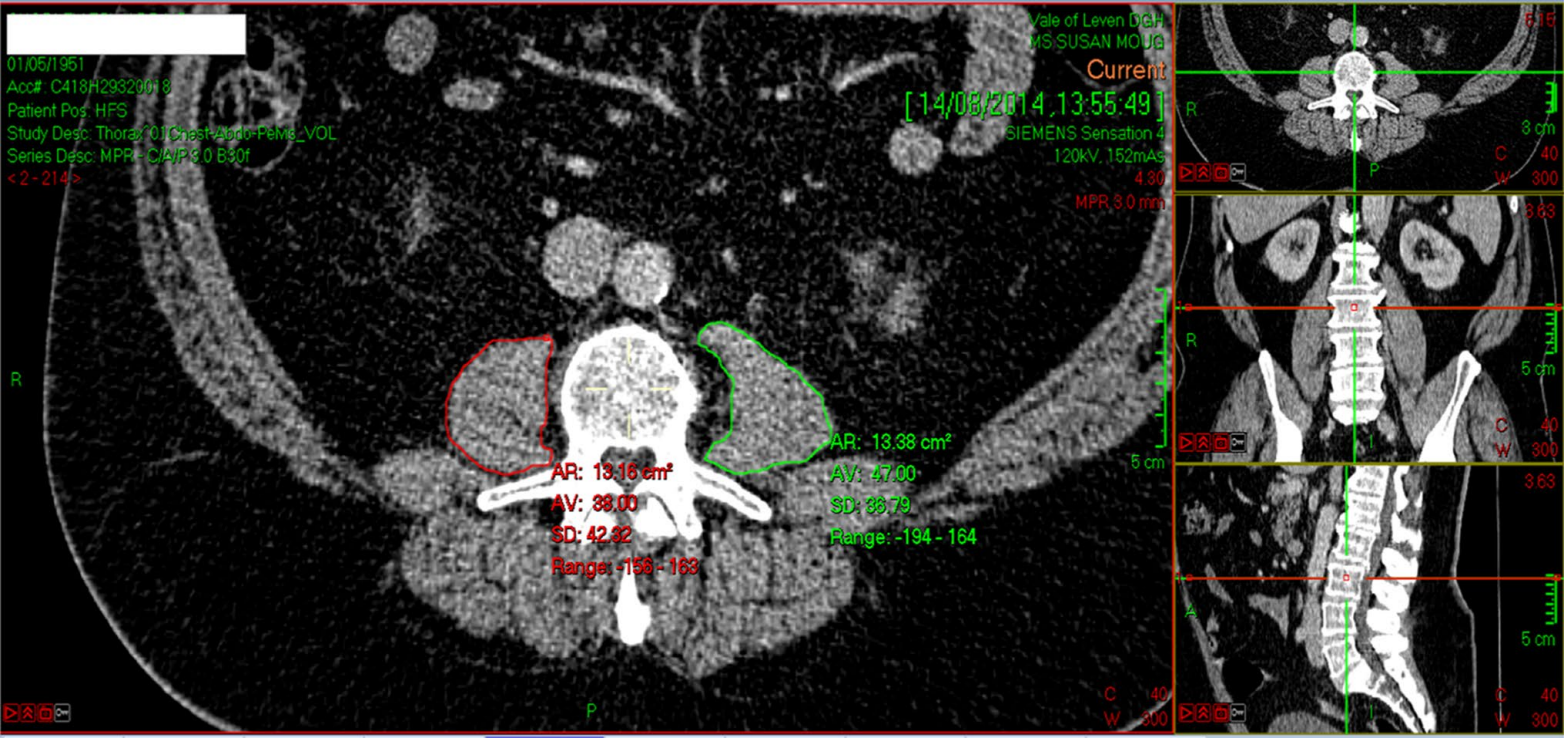
CT image at the level of the L3 vertebra, demonstrating the method of outlining the left (green) and right (red) psoas muscle and measuring their respective areas

To measure sarcopenia in each patient, the total cross-sectional area of the psoas muscles (total psoas area, TPA) was measured using a manual technique at the level of the L3 vertebra on preoperative CT (median interval between CT scan and surgery was 38 days, range 2–119 days) [22]. To ensure standardisation, the exact level of measurement was defined as the CT slice in which both L3 transverse processes were maximally in view. Area was measured using a free-hand drawing technique on Picture Archiving and Communication System (PACS) software (Fig. 1). The outline of each individual psoas muscle was traced, the area of each calculated, and summated to provide the TPA (mm^2^). The TPA was then standardised for patient height using the formula: TPA (mm^2^)/height (m^2^). This provided the total psoas index (TPI) for each patient.

For the purposes of this study, the threshold values used for the diagnosis of sarcopenia are the same as those used by Prado et al. in their widely cited 2008 paper [23]: 524 mm^2^/m^2^ for males and 385 mm^2^/m^2^ for females. All individuals with a TPI below this threshold for their gender were classified as sarcopenic. For the purposes of this study, sarcopenia is defined as an absolute variable; patients were either sarcopenic or non-sarcopenic.

To ensure reliability of our technique, 20 scans were randomly selected and measured for  TPI  by blindly trained investigators to allow calculation of inter- and intra-class correlation coefficients (ICCC). The ICCC values for inter- and intra-class reliability were 0.94 and 0.99, respectively (values close to 1 indicate excellent agreement).

### Statistical analysis

Relevant variables were transcribed to SPSS version 21 [24] to enable statistical analysis. Descriptive statistics were used to characterise the patient population, with continuous variables summarised by median and interquartile range and categorical variables presented in tabulated form with percentages. Continuous variables were tested using non-parametric methods.

To determine the primary outcome of the influence of sarcopenia on survival at 1 year, survival analysis using log-rank testing was performed. Further exploration of variables influencing mortality was performed using univariate analysis and a Cox proportional hazards model. The level of significance was set at 5%.

## Results

### Patient demographics, physiological and pathological characteristics

**Fig. 2:**
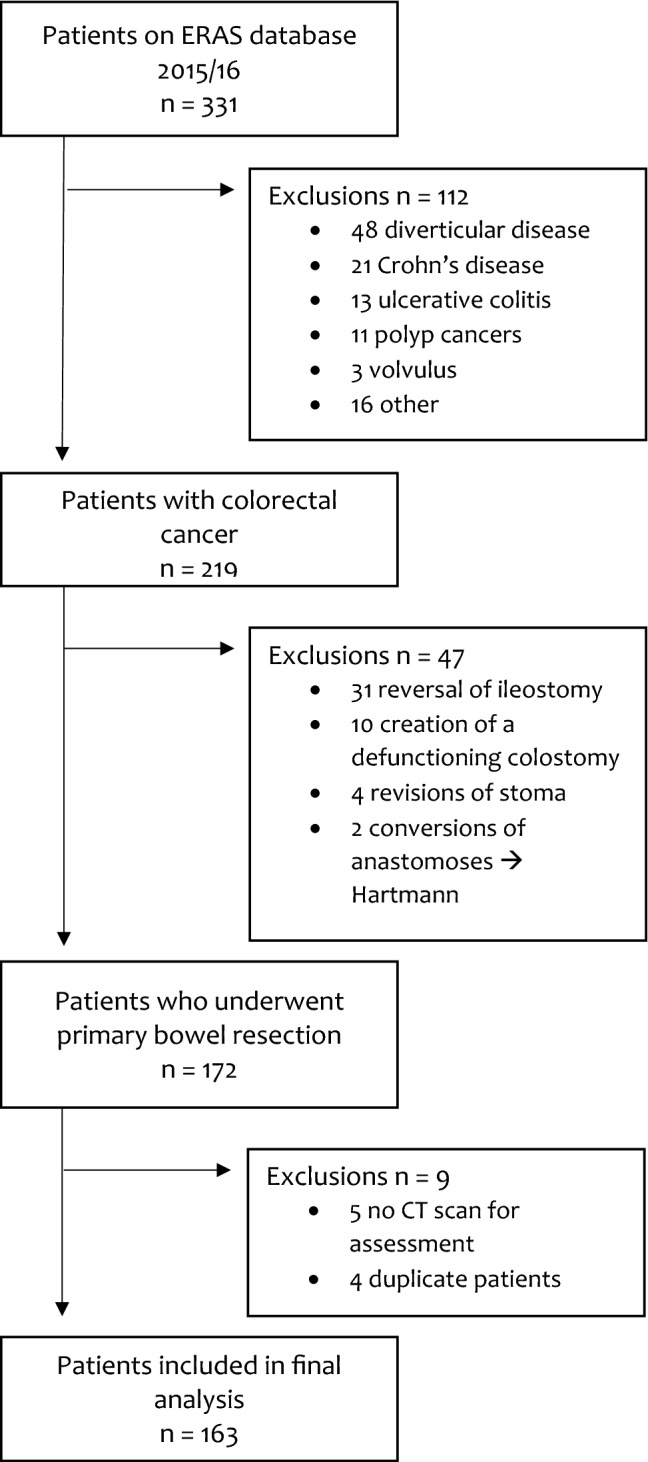
Patient flowchart for the study and reasons for exclusion of individuals. *ERAS* enhanced recovery after surgery

Over the study period, 331 patients had elective colorectal surgery, with 163 (49.2%) eligible for inclusion in the study (Fig. 2). The median age of the cohort was 70 years (IQR 61–75) and 99 (60.7%) were male. The majority of surgery was for patients with rectal cancer who had an ASA of 2 and 112 (68.7%) were overweight or obese. One hundred and forty-nine (91.4%) had a pathological R0 resection with only 8.6% of patients having a major complication leading to a median length of the hospital stay of 8 days (IQR 6–12) [Table 1]. Eleven patients had stage 4 tumours, however, these patients had lung/liver metastases that were considered resectable and so they were operated on with curative intent.

**Table 1:** Baseline demographics, clinical, and pathological characteristics of the patients underdoing elective surgery for colorectal cancer

Characteristic	*n* (%)
Sex	
Male	99 (60.7)
Female	64 (39.3)
Median age, years (IQR)	
	70 (61–75)
Age category, years	
< 65	57 (35)
65–74	59 (36.2)
≥ 75	47 (28.8)
BMI category kg/m^2^	
Underweight	4 (2.5)
Normal	47 (28.8)
Overweight	67 (41.1)
Obese	45 (27.6)
ASA grade	
1	4 (2.5)
2	101 (62)
3	56 (34.4)
4	2 (1.2)
TNM stage	
0	8 (4.9)
1	34 (20.9)
2	57 (35)
3	53 (32.5)
4	11 (6.7)
Site of cancer	
Colon	72 (44.2)
Rectum	91 (55.8)
Procedure	
Right hemicolectomy	52 (31.9)
Left hemicolectomy or sigmoid colectomy	15 (9.2)
Total or sub-total colectomy	6 (3.7)
Anterior resection	65 (39.9)
APER	25 (15.3)
Resection margin	
R0	149 (91.4)
R1	14 (8.6)
In-hospital complication	
Yes	91 (55.8)
No	72 (44.2)
Major in-hospital complication*	
Yes	14 (8.6)
No	149 (91.4)
Median length of stay (IQR), days	
	8 (6–12)
Re-admitted within 30 days	
Yes	21 (12.9)
No	142 (87.1)
Sarcopenic	
Yes	32 (19.6)
No	131 (80.4)

*Complications classified as 3 or greater on the Clavien–Dindo scale

*ASA* American Society of Anesthesiologists, *AJCCTNM* American Joint Committee on Cancer TNM stage, *BMI* body mass index

### Sarcopenia

**Fig. 3:**
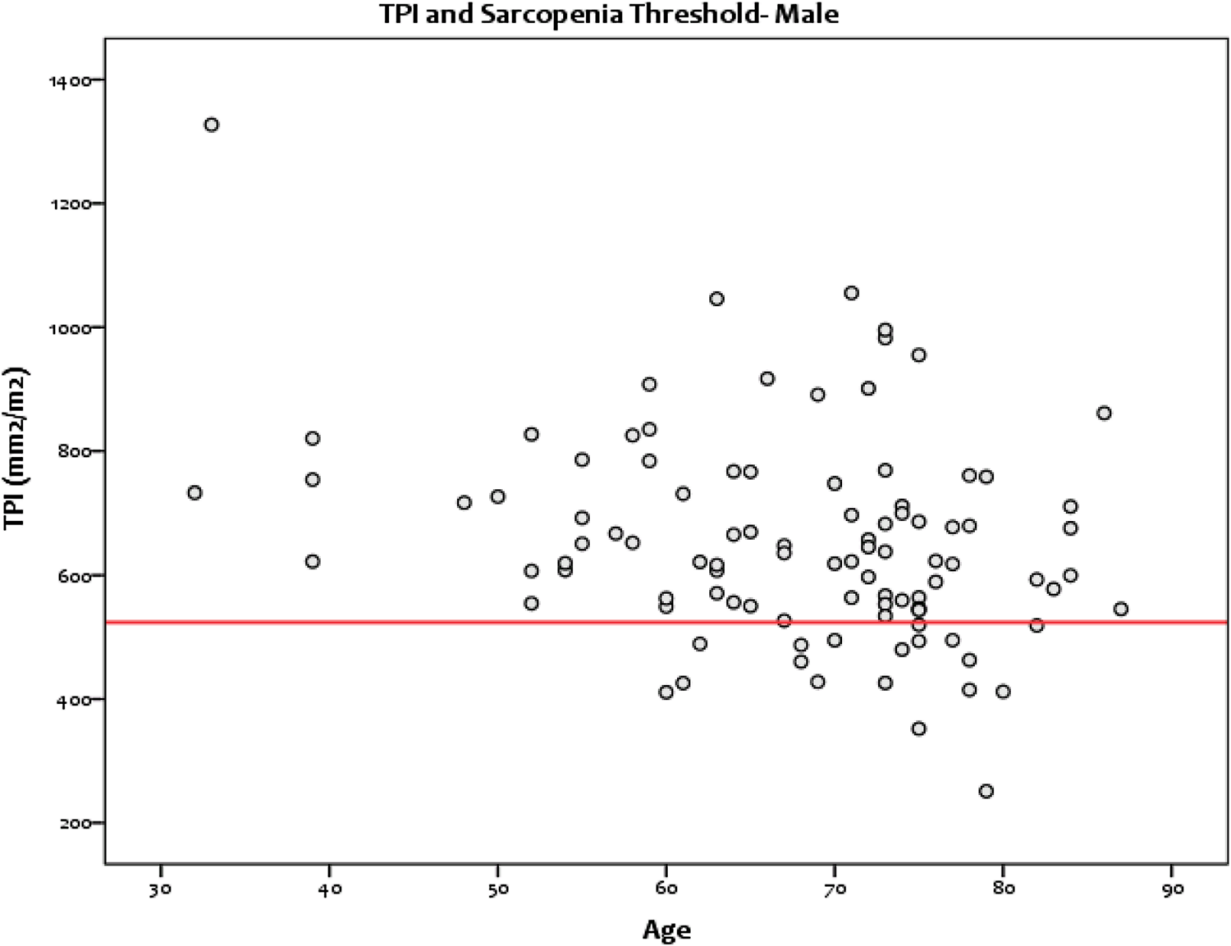
Scatterplot showing the spread of TPI values for male patients and the threshold for sarcopenia

**Fig. 4:**
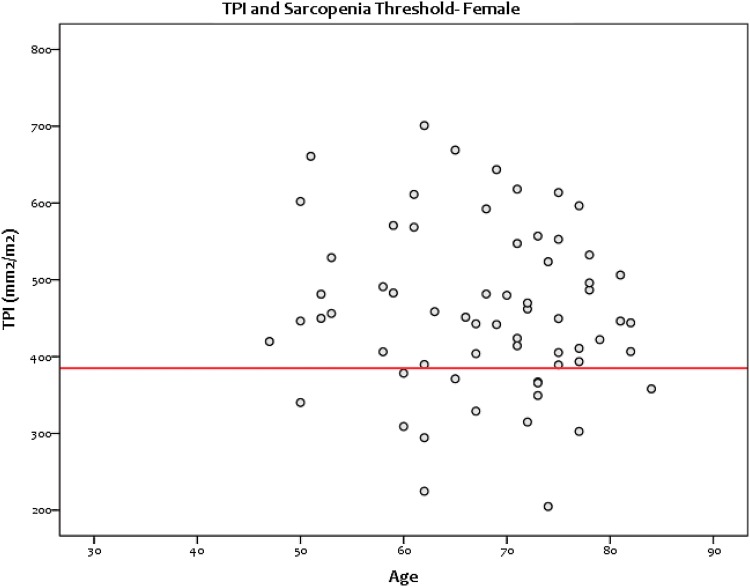
Scatterplot showing the spread of TPI values for female patients and the threshold for sarcopenia

**Fig. 5:**
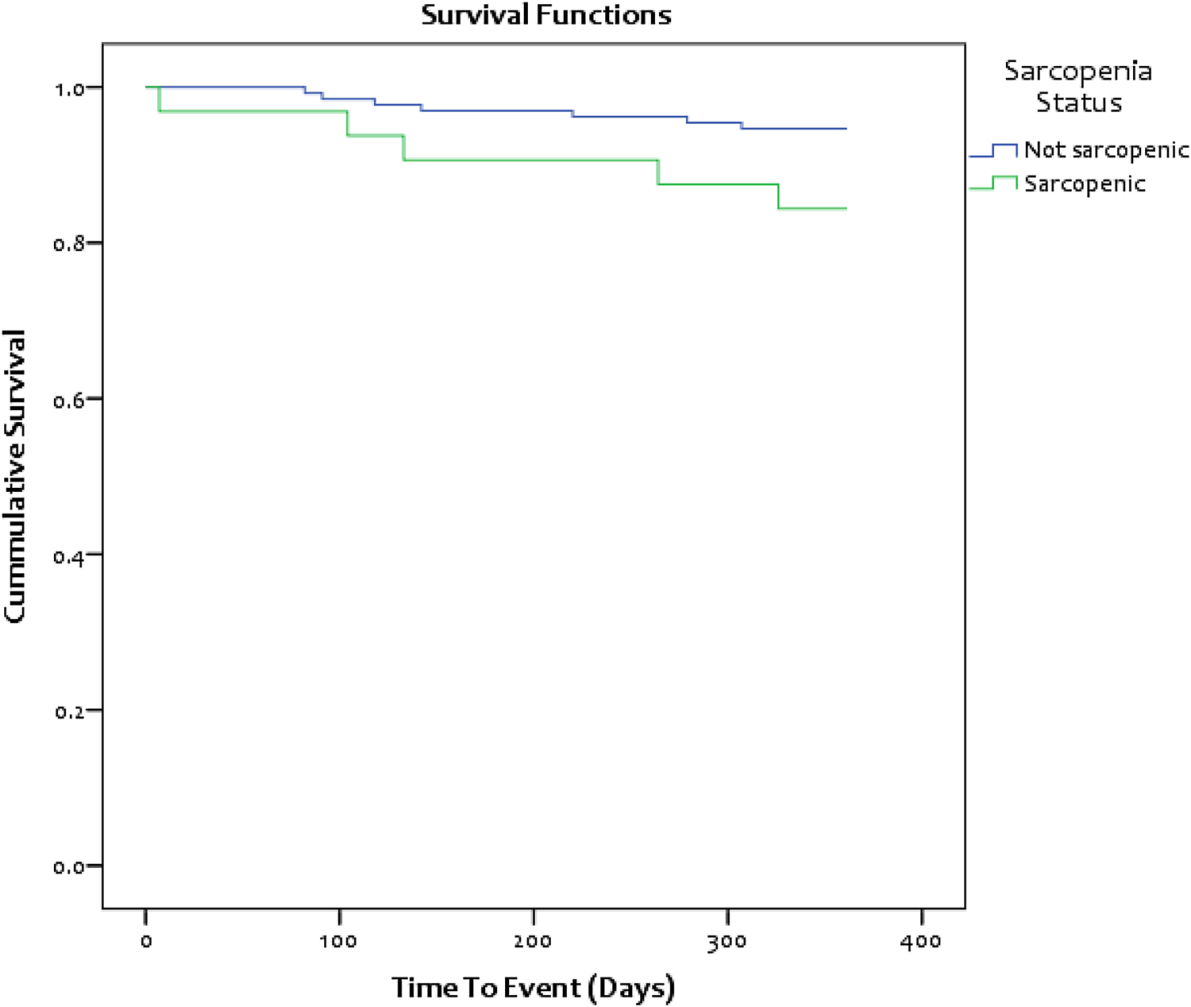
Comparison of 1-year mortality between colorectal cancer patients with sarcopenia and those without sarcopenia

Overall, 32 (19.6%) patients having elective colorectal cancer surgery were sarcopenic: 18/99 males (18.2%) (Fig. 3) and 14/64 females (21.9%) (Figs. 3, 4). The characteristics of the sarcopenic and non-sarcopenic groups are compared in Table 2. Significant differences were noted between the two groups in the categories: BMI (*p* = 0.007), 30-day mortality (*p* = 0.042) and 1-year mortality (*p* = 0.046). Here, the sarcopenic patients were more likely to be classified as underweight and have increased postoperative mortality at 30 days and 1 year [Fig. 5].

**Table 2:** Demographics, clinical, and pathological characteristics of patients with sarcopenia compared to patients without sarcopenia

	Sarcopenic (*n* = 32)	Non-sarcopenic (*n* = 131)	*p* value
Sex			
Male	18 (56.3)	81 (61.8)	0.350
Female	14 (43.8)	50 (38.2)	
Age category, years			
< 65	8 (25.0)	49 (37.4)	0.412
65–74	13 (40.6)	46 (35.1)	
≥ 75	11 (34.4)	36 (27.5)	
BMI category kg/m^2^			
Underweight	3 (9.4)	1 (0.8)	0.007*
Normal	13 (40.6)	34 (26.0)	
Overweight	11 (34.4)	56 (42.7)	
Obese	5 (15.6)	40 (30.5)	
Median ASA (SD)			
	2 (0.56)	2 (0.55)	0.542
TNM stage			
0	3 (9.4)	5 (3.8)	0.194
1	3 (9.4)	31 (23.7)	
2	12 (37.5)	45 (34.4)	
3	12 (37.5)	41 (31.3)	
4	2 (6.3)	9 (6.9)	
Site of cancer			
Colon	17 (53.1)	55 (42.0)	0.174
Rectum	15 (46.9)	76 (58.0)	
Resection margin			
R0	30 (93.8)	119 (90.8)	0.598
R1	2 (6.3)	12 (9.2)	
In-hospital complication			
Yes	14 (43.8)	73 (55.7)	0.957
No	18 (56.3)	60 (44.3)	
Major in-hospital complication			
Yes	2 (6.3)	12 (9.2)	0.598
No	30 (93.8)	119 (90.8)	
Median length of stay (IQR), days			
	8 (6–12)	8 (6–12)	0.567
Re-admitted within 30 days			
Yes	3 (9.4)	18 (13.7)	0.509
No	28 (90.6)	113 (86.3)	
Alive at 30 days			
Yes	31 (96.9)	131 (100)	0.042*
No	1 (3.1)	0 (0)	
Alive at 90 days			
Yes	31 (96.9)	130 (0.8)	0.277
No	1 (3.1)	1 (99.2)	
Alive at 1 year			
Yes	27 (84.4)	124 (94.7)	0.046*
No	5 (15.6)	7 (5.3)	

Complications classified as 3 or greater on the Clavien–Dindo scale

*ASA* American Society of Anesthesiologists, *AJCCTNM* American Joint Committee on Cancer TNM stage, *BMI* body mass index

*P < 0.05: level of significance

### Survival at 1 year

For the whole patient cohort, univariate analysis found sarcopenia (*p* = 0.043), tumour stage (*p* = 0.018), ASA grade (*p* = 0.016) and major complications (*p* = 0.021) to be significantly associated with survival at 1 year. Multivariable analysis revealed that only ASA grade and tumour stage were significantly independent predictors of mortality at 1 year (*p* = 0.042 and *p* = 0.007, respectively) (Table 3).

**Table 3:** Univariate and multivariable analyses of factors influencing survival at 1 year in patients who have undergone elective surgery for colorectal cancer

	Univariate analysis	Multivariable analysis^a^		
Variable	Log-rank *p* value	Hazard ratio	95% CI	*p* value
Sarcopenia	0.043	2.233	0.665–7.504	0.194
TNM stage	0.018	2.609	1.307–5.208	0.007
ASA	0.016	2.861	1.037–7.892	0.042
Major complication	0.021	–	–	–
BMI category	0.225	–	–	–
Age group	0.546	–	–	–
Gender	0.418	–	–	–

*ASA* American Society of Anesthesiologists, *BMI* body mass index

^a^Included preoperative variables that had been identified as significant in univariate analysis

## Discussion

This study demonstrated that sarcopenia is prevalent, occurring in nearly one-fifth of the patients having elective colorectal cancer surgery. In addition to confirming that sarcopenia places patients at greater risk of postoperative complications, this work has found sarcopenia also negatively influences survival at 1 year after curative surgery.

These results have strengths over the previous publications looking at 1 year survival. It is prospective and contains both colon and rectal cancer patient populations, not just rectal [18]. In addition, two of the previous studies assessed sarcopenia in Asian populations, and members of these populations have accepted differences in lifestyle and body habitus when compared to the western population [18, 19]. For example, in the paper by Choi et al., mean age of the patients was 61.3 years and mean BMI was 23.8 kg/m^2^. These figures are both significantly lower than in the cohort of patients included in this study. Overall, our findings are more applicable to the western colorectal cancer patients, adding to the evolving evidence that sarcopenia is a negative prognostic factor for patients having elective gastrointestinal oncological surgery, and more specifically, colorectal resection.

The mechanism underlying the significant relationship between sarcopenia and 1-year mortality requires discussion. It may be indicative of the presence of sub-clinical synchronous metastasis, which is subsequently picked up during follow-up after surgery. If this is the case, then sarcopenia may highlight patients who would benefit from more extensive staging investigations, such as positron emission tomography (PET) scanning. This may lead to the consideration of preoperative chemotherapy and/or more frequent clinical and radiological follow-up with the aim of improving outcomes. An alternative explanation is that sarcopenia indicates that patients have poorer physical capacity, making them less resilient to the physiological stresses of surgery and more at risk of complication [15]. Indeed, the relationship between sarcopenia and frailty is well documented [25]. This raises the possibility that, unlike other preoperative prognostic markers (e.g. TNM staging), sarcopenia could be modified via prehabilitation. This individualised physical activity-centred intervention is delivered in the period between diagnosis and the commencement of treatment for cancer and has been shown to be feasible, safe and reduce postoperative complications in patients treated for colorectal cancer [26, 27], although may not always produce improvements in fitness. Furthermore, a recent systematic review has demonstrated that the positive effects of prehabilitation can be seen after as little as 2 weeks [28]. Therefore, the pretreatment measurement of sarcopenia could offer an effective way of selecting patients who would benefit the most from such targeted, individualised prehabilitation [29]. Individualised prehabilitation programmes will require adequate funding to introduce; however, we do not expect interventions to be particularly costly, and would hope that the institution of prehabilitation would lead to savings in resources due to reduced morbidity and shortened stays in hospital.

The strengths of our study were that the data were collected prospectively and are reviewed centrally on a monthly basis, minimising the likelihood of any inaccuracy in the measurement or recording of variables. The method used to measure sarcopenia is simple and easy to learn, low cost, valid, and requires no investment from healthcare providers, meaning it could be seamlessly integrated into the care pathway for colorectal cancer patients. This is in contrast to other methods of skeletal muscle measurement that use costly specialist software packages that require additional training.

It is important to acknowledge several limitations of this work. First, this is a single-centre study and future work should be multi-centred to allow greater number of patients with colorectal cancer to be included. A multi-centre study would also allow the inclusion of a more ethnically diverse population and subsequent subgroup analysis. Second, although all scans were performed within 4 months of surgery, it is possible that the skeletal muscle mass could have changed before surgery. Furthermore, the use of predefined cutoffs for sarcopenia has been defined in populations, which are not necessarily homogenous to the population included in this study. For example, the Prado sarcopenia cutoffs used in this study were defined in an obese population in Canada, with tumours of the respiratory and gastrointestinal tracts. However, using predefined cutoffs is preferable to arbitrarily concluding that patients in the lowest quartile for muscle mass are sarcopenic. Lastly, 30-day mortality was related to sarcopenia in this study. However, this finding was driven by a single death in the cohort and should be interpreted with caution.

## Conclusions

This study has found sarcopenia to be prevalent in colorectal cancer patients having elective surgery, resulting in poorer long-term survival. CT measurement of total psoas mass is a valid and simple technique for diagnosing sarcopenia that could be used to augment existing methods of patient-risk stratification prior to surgery. Such sarcopenic patients could then undergo targeted strategies such as prehabilitation, to improve both their short- and long-term outcomes.
